# New Orientia tsutsugamushi strain from scrub typhus in Australia.

**DOI:** 10.3201/eid0404.980416

**Published:** 1998

**Authors:** D M Odorico, S R Graves, B Currie, J Catmull, Z Nack, S Ellis, L Wang, D J Miller

**Affiliations:** James Cook University, Townsville, Queensland, Australia.

## Abstract

In a recent case of scrub typhus in Australia, Orientia tsutsugamushi isolated from the patient's blood was tested by sequence analysis of the 16S rDNA gene. The sequence showed a strain of O. tsutsugamushi that was quite different from the classic Karp, Kato, and Gilliam strains. The new strain has been designated Litchfield.


### New *Orientia tsutsugamushi* Strain from Scrub Typhus in Australia

**Dimitri M. Odorico,* Stephen R. Graves,† Bart Currie,‡ Julian Catmull,* Zoltan Nack,† Sharon Ellis,† Ling Wang,† and David J. Miller***

*James Cook University, Townsville, Queensland, Australia; †The Geelong Hospital, Geelong, Victoria, Australia; and ‡Royal Darwin Hospital, Casuarina, Northern Territory, Australia

In a recent case of scrub typhus in Australia, *Orientia tsutsugamushi* isolated from the patient's blood was tested by sequence analysis of the 16S rDNA gene. The sequence showed a strain of *O. tsutsugamushi* that was quite different from the classic Karp, Kato, and Gilliam strains. The new strain has been designated Litchfield.

Scrub typhus has been recognized as an important cause of fever among inhabitants of tropical Queensland (northeast Australia) for the past 60 years (1-6). All three groups of rickettsial diseases occur in Australia (Figure). 1) Spotted fever group (SFG): i) *Rickettsia australis* (Queensland tick typhus) occurs along the eastern side of the continent to the east of the Great Dividing Range (7,8); ii) *R. honei* (Flinders Island spotted fever) is only now recognized on Flinders Island—a small island off the southeast corner of Australia (9,10); iii) an unidentified SFG rickettsial infection in Tasmania, a large island state to the southeast of the Australian mainland, has been identified through serologic evidence, but no isolate has been obtained. 2) Typhus group (TG): *R. typhi* (murine typhus) occurs throughout Australia, with foci recognized in southwest Australia, southcentral Australia (first described by Hone [11] in Adelaide, South Australia), and central Queensland (northeast Australia). 3) Scrub typhus: *Orientia tsutsugamushi* infection, well recognized in tropical coastal Queensland, has probably long been endemic in this area. As Europeans gradually settled the area, the etiologies of numerous febrile illnesses were determined. Serologic evidence (the Weil-Felix serologic test) of scrub typhus became available (1-3) and later isolates of *O. tsutsugamushi* were made by inoculating mice with patients' blood (4). A serologic analysis of the Queensland strains of *O. tsutsugamushi* suggested that most strains were Karp-like (12). (The Karp strain was isolated from Papua New Guinea [13].) The geographic distribution of scrub typhus in the rest of Australia is less clear. The disease was not considered to occur west of coastal Queensland. However, in the tropical region of the Northern Territory, six possible cases from 1937 to 1939 and one in 1957 were reported (all presumptively diagnosed). Since 1990 , however, nine cases of scrub typhus have been reported in Litchfield Park, a discrete area of rain forest in the

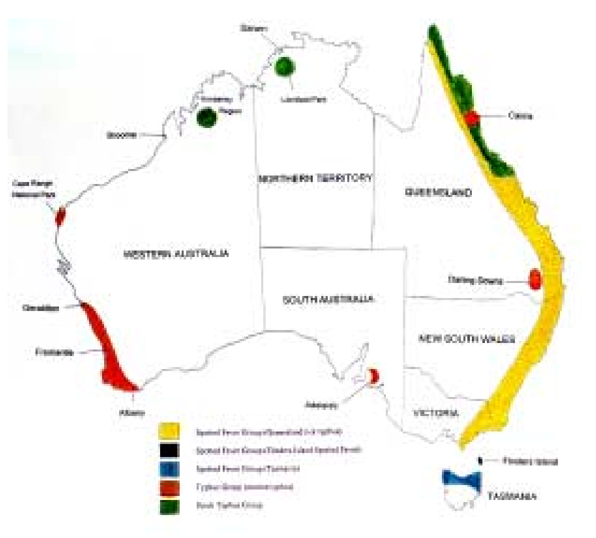

Figure. Geographic distribution of rickettsial diseases in Australia.

Address for correspondence: Stephen R. Graves, Australian Rickettsial Reference Laboratory, The Geelong Hospital, Geelong, Victoria 3220, Australia; fax: 61-3-5221-2232; e-mail:stepheng@gh.vic.gov.au.

Northern Territory opened as a park in 1986 (16). All patients had eschars, and all cases were confirmed by specific *O. tsutsugamushi* serologic tests. A case of scrub typhus was also reported from a similar rain forest pocket in the tropical Kimberley region of northwestern Australia (17).

### Case Report

In mid-August 1996, a 38-year old man working on the construction of a tourist path in the rain forest fringe of Litchfield Park (15) became ill with fever, sweats, headache, sore throat, cough, lethargy, and confusion during his second week on the job. His condition worsened over at least a week. He eventually received amoxycillin and clavulanate from a local medical practitioner but contracted diarrhea and was admitted to Royal Darwin Hospital with fever, rigors, but no evident focus of infection. A diagnosis of septicemia was made, and he was started on ceftriaxone and gentamicin.

Over the next day, he became increasingly confused, and his fever persisted. He became hypotensive, hypoxemic, and oliguric and was transferred to the intensive care unit. On day 3 after admission, he required fluid loading, inotrope therapy, intubation, and ventilation. At that time, his work history was obtained from relatives, and a 6-mm sore with a necrotic dark center was noted on his upper right buttock. Scrub typhus was suspected, and he was given intravenous doxycycline. However, his condition continued to deteriorate with hypotension, renal failure, and adult respiratory distress syndrome. Mucosal and gastrointestinal bleeding developed, and the patient died 6 days after admission.

### Laboratory Investigation

### Rickettsial Serologic Tests

Paired sera, 6 days apart, showed a greater than fourfold rise in antibody titer to *O. tsutsugamushi* by microimmunofluorescence (15). Sera did not react to SFG rickettsiae or TG rickettsiae. All three classic strains of *O. tsutsugamushi* (Karp, Kato, Gilliam) reacted with the patient's serum, at titers of >1/1024 (negative <1/64).

### Mouse Inoculation

Three female outbred white mice were inoculated with blood taken from the patient 4 days before he died. The mice were inoculated intraperitoneally with 0.1 ml, 0.2 ml, and 0.5 ml of whole blood in EDTA. The mice were housed in a flexible film isolator in a high security microbiology laboratory at the Australian Animal Health Laboratory, Geelong, Australia. None of the mice died.

Subsequently three additional mice were inoculated with very concentrated preparations of the *O. tsutsugamushi* strain isolated from the patient; none died. This strain of *O. tsutsugamushi* appears not to be virulent for mice despite being highly virulent for the patient. No attempt was made to isolate the strain from mouse organs.

Seroconversion of the inoculated mice to *O. tsutsugamushi* was confirmed by microimmunofluorescence.

### Rickettsial DNA Detection by Polymerase Chain Reaction (PCR)

One ml of the patient's blood, taken 4 days before death, was lysed; DNA was extracted (18); and part of the 56kDa-type specific antigen (an outer membrane protein) gene was amplified by PCR. Primers 'a' (5'-TACATTAGCTGCGGGTATGACA-3') and 'b' (5'-CCAGCATAATTCTTCAACCAAG-3') were used as previously described (19), except that a nested procedure was not used. The PCR product was electrophoresed on agarose gel, stained with ethidium bromide, and detected by UV fluorescence. Positive and negative controls were run with the unknown samples. The PCR product from the patient's blood had the same molecular weight as a PCR product from a known *O. tsutsugamushi* strain.

### Tissue Culture Infection

Three 25-$cm^2$ flasks containing a confluent monolayer of Vero cells were infected with whole blood (in EDTA) from the patient (0.1 ml, 0.5 ml, and 1.0 ml, respectively). After sitting at 35°C for 4 hours without centrifugation, the blood was removed, and the monolayers were washed twice with Hank's balanced salt solution. The medium used for culture was RPMI 164O with 10% fetal calf serum (heat-inactivated) at 35°C (without $CO_2$). Cultures were examined weekly for abnormalities, and the pH was adjusted as necessary. On day 60 postinfection, a 3+ cytopathogenic effect was observed in one flask, with most Vero cells floating in the supernatant. These cells were tested for bacterial contamination (Gram stain–negative; growth on horse blood agar–negative) and for rickettsiae (positive for Giminez stain; positive for scrub typhus, as determined by immunofluorescence staining with convalescent-phase human serum; and positive as determined by PCR for 56 kDa antigen gene). The isolated strain was subcultured and grown for DNA extraction.

### 16S rDNA Sequencing

The 16S rDNA was amplified by PCR with primers A and H*(20), and the products were cloned into pGEM-T (Promega). The complete nucleotide sequences of both strands of several clones were determined by using internal primers (20,21); sequencing reactions were analyzed on an ABI 310 Genetic Analyser by using the ABI Big-Dye chemistry. The sequence has been submitted to GenBank under the accession number AF062074.

Comparison with the databases confirmed that the sequence from the novel isolate was *O. tsutsugamushi* but indicated that it differed greatly from the four type strains (Karp, Kuroki, Gilliam, Kato). The novel isolate has five unique base substitutions in the 16S rDNA and thus differs more from the reference strains than they do from each other. On this basis, it was designated a new strain and was called Litchfield.

The Litchfield strain of *O. tsutsugamushi* is the first isolated in culture from the Northern Territory of Australia, although previous isolates have been made from Queensland (4,12). *O. tsutsugamushi* has probably been present in the mites and native mammals of northern Australia for millennia. One of the most common mite vectors, *Leptotrombidium deliense,* has been detected on several species of native rat in Litchfield Park (22), although *O. tsutsugamushi* has not yet been isolated from native mammals from the Northern Territory. In Queensland, however, *O. tsutsugamushi* has been isolated from native mammals and mites (23-25).

The emergence of scrub typhus in Litchfield Park in the Northern Territory of Australia is probably due to increasing contact between humans and this scrub and rain forest environment and its animals. Tourism is a major industry in Australia, and increasing numbers of visitors are traveling to remote parts to experience the tropical "out-back." Thus, public health officials and local health providers need to be aware of foci of endemic diseases in remote parts of Australia. One problem with rickettsial diseases is that they do not respond to ß-lactam antibiotics (e.g., penicillins and cephalosporins) because of their unusual bacterial cell wall or to aminoglycoside antibiotics (e.g., gentamicin) because of their intracellular location. Treatment of patients with undiagnosed sepsis in Australian hospitals is very likely to involve the use of a cephalosporin plus gentamicin, a combination that is not effective against scrub typhus or any other rickettsial disease. The travel history of a febrile patient is essential if tropical infections are to be included in the differential diagnosis of febrile illness.

The 16S rDNA evolves relatively slowly. Hence, despite geographic proximity, the key difference between the sequences from the Karp strain (isolated from a patient in New Guinea) and the Litchfield isolate implies a long period of isolation between these strains. Few general conclusions can be drawn from one sequence for a single Australian isolate. However, the prediction is that at least some Australian strains of *O. tsutsugamushi* will differ substantially from type strains.

Dr. Odorico is a molecular biologist with a broad range of interests, including molecular phylogenetics. His present research involves the functional analysis of eukaryotic promoters by use of site-directed mutagenesis and reporter gene techniques.

### Acknowledgment

We thank Letka Kociska and Lyn Merkelbach for assistance with preparation of this report.

### References

1. Langan AM, Mathew RY. The establishment of "Mossman," "Coastal" and other previously unclassified fevers of north Queensland as endemic typhus. Med J Aust 1935; Aug 3:145-8.
2. Unwin ML. "Coastal fever" and endemic tropical typhus in north Queensland: recent investigation, clinical and laboratory findings. Med J Aust 1935; Sept 7:303-8.
3. Heaslip WG. Tsutsugamushi fever in north Queensland, Australia. Med J Aust 1941; March 29:380-92.
4. Carley JG, Doherty RL, Derrick EH, Pope JH, Emanuel ML, Ross CJ. The investigation of fevers in north Queensland by mouse inoculation with particular reference to scrub typhus. Australasian Annals of Medicine 1955;4:91-9.
5. Doherty RL. A clinical study of scrub typhus in north Queensland. Med J Aust 1956; Aug 11:212-20.
6. Derrick EH. The incidence and distribution of scrub typhus in north Queensland. Australasian Annals of Medicine 1961;10:256-67.

7. Andrew R, Bonnin JM, Williams S. Tick typhus in Queensland. Med J Aust 1946; Aug 24;253-8.
8. Sexton DJ, Dwyer B, Kemp R, Graves S. Spotted fever group rickettsial infections in Australia. Reviews of Infectious Diseases 1991;13:876-86.
9. Stewart RS. Flinders Island spotted fever: a newly recognised endemic focus of tick typhus in Bass Strait. Part 1. Clinical and epidemiological features. Med J Aust 1991;154:94-9.
10. Graves SR, Dwyer BD, McColl D, McDade JE. Flinders Island spotted fever: a newly recognised endemic focus of tick typhus in Bass Strait. Part 2. Serological investigations Med J Aust 1991;154:99-104.
11. Hone FS. A series of cases closely resembling typhus fever. Med J Aust 1922; Jan 7:1-13.
12. Shirai A, Campbell RW, Gan E, Chan TC, Huxsoll DL. Serological analysis of *Rickettsia tsutsugamushi* isolates from north Queensland. The Australian Journal of Experimental Biology and Medical Science 1982;60:203-5.
13. Derrick EH, Brown HE. Isolation of the Karp strain of *Rickettsia tsutsugamushi*. Lancet 1949;July 23:150-1.
14. Cook GC, editor. Manson's tropical diseases. 20th ed. Philadelphia: WB Saunders Company Ltd.; 1996. p. 809.
15. Currie B, Lo D, Marks P, Lum G, Whelan P, Krause V. Fatal scrub typhus from Litchfield Park, Northern Territory. Commun Dis Intell 1996;20;420-1.
16. Currie B, O'Connor L, Dwyer B. A new focus of scrub typhus in tropical Australia. Am J Trop Med Hyg 1993;49:425-9.
17. Quinlan ML, Chappell T, Golledge CL. Scrub typhus in Western Australia. Commun Dis Intell 1993;17:570-1.
18. Wyman AR, White R. A highly polymorphic focus in human DNA. Proc Natl Acad Sci U S A 1980;77;6754-8.
19. Kawamori F, Akiyama M, Sugieda M, Kanda T, Akahane S, Yamamoto S, et al. Two-step polymerase chain reaction for diagnosis of scrub typhus and identification of antigenic variants of *Rickettsia tsutsugamushi*. J Vet Med Sci 1993;55:749-55.
20. Edwards U, Rogall T, Blocker H, Emde M, Bottger EC. Isolation and direct complete nucleotide determination of entire genes. Characterisation of a gene coding for 16S ribosomal RNA. Nucleic Acids Res 1989;17(19):7843-53.
21. Wilson KH, Blitchington RB, Greene RC. Amplification of bacterial 16S ribosomal DNA with polymerase chain reaction. J Clin Microbiol 1990;28:1942-6.
22. Bell PJ, Whelan PI. A scrub typhus vector *Leptotrombidium deliense* (Walch) (Acari: Trombiculidae) on rats in Litchfield Park, Northern Territory, Australia. Journal of the Australian Entomological Society 1993;32:207-8.
23. Cook I, Scott W, Campbell RW. Scrub typhus and other infections in north Queensland animals. Trans R Soc Trop Med Hyg 1967;61:343-50.
24. Campbell RW, Domrow R. Rickettsioses in Australia: isolation of *Rickettsia tsutsugamushi* and *R. australis* from naturally infected anthropods. Trans R Soc Trop Med Hyg 1974;68:397-402.
25. Glazebrook JS, Campbell RSF, Hutchinson GW, Stallman ND. Rodent zoonoses in North Queensland, the occurrence and distribution of zoonotic infections in north Queensland rodents. The Australian Journal of Experimental Biology and Medical Science 1978;56:147-56.
